# The *Pseudomonas aeruginosa* biofilm matrix and cells are drastically impacted by gas discharge plasma treatment: A comprehensive model explaining plasma-mediated biofilm eradication

**DOI:** 10.1371/journal.pone.0216817

**Published:** 2019-06-24

**Authors:** Juliana Soler-Arango, Cecilia Figoli, Giuliana Muraca, Alejandra Bosch, Graciela Brelles-Mariño

**Affiliations:** 1 Biofilm Eradication Laboratory, Center for Research and Development of Industrial Fermentations, Consejo Nacional de Investigaciones Científicas y Técnicas (CINDEFI, CCT-LA PLATA-CONICET), Facultad de Ciencias Exactas, Universidad Nacional de La Plata, La Plata, Argentina; 2 Bioespectroscopy Laboratory, Center for Research and Development of Industrial Fermentations, Consejo Nacional de Investigaciones Científicas y Técnicas (CINDEFI, CCT-LA PLATA-CONICET), Facultad de Ciencias Exactas, Universidad Nacional de La Plata, La Plata, Argentina; Institute of Materials Science, GERMANY

## Abstract

Biofilms are microbial communities encased in a protective matrix composed of exopolymeric substances including exopolysaccharides, proteins, lipids, and extracellular DNA. Biofilms cause undesirable effects such as biofouling, equipment damage, prostheses colonization, and disease. Biofilms are also more resilient than free-living cells to regular decontamination methods and therefore, alternative methods are needed to eradicate them. The use of non-thermal atmospheric pressure plasmas is a good alternative as plasmas contain reactive species, free radicals, and UV photons well-known for their decontamination potential against free microorganisms. *Pseudomonas aeruginosa* biofilms colonize catheters, indwelling devices, and prostheses. Plasma effects on cell viability have been previously documented for *P*. *aeruginosa* biofilms. Nonetheless, the effect of plasma on the biofilm matrix has received less attention and there is little evidence regarding the changes the matrix undergoes. The aim of this work was to study the effect plasma exerts mostly on the *P*. *aeruginosa* biofilm matrix and to expand the existing knowledge about its effect on sessile cells in order to achieve a better understanding of the mechanism/s underlying plasma-mediated biofilm inactivation. We report a reduction in the amount of the biofilm matrix, the loss of its tridimensional structure, and morphological changes in sessile cells at long exposure times. We show chemical and structural changes on the biofilm matrix (mostly on carbohydrates and eDNA) and cells (mostly on proteins and lipids) that are more profound with longer plasma exposure times. We also demonstrate the presence of lipid oxidation products confirming cell membrane lipid peroxidation as plasma exposure time increases. To our knowledge this is the first report providing detailed evidence of the variety of chemical and structural changes that occur mostly on the biofilm matrix and sessile cells as a consequence of the plasma treatment. Based on our results, we propose a comprehensive model explaining plasma-mediated biofilm inactivation.

## Introduction

Biofilms are surface-associated microbial communities encased in a protective matrix composed of exopolymeric substances (EPS) including exopolysaccharides, proteins, lipids, and extracellular DNA (eDNA). Biofilm formation takes place in several steps starting with free-living (planktonic) bacteria recognizing a surface, and ending in the development of a mature biofilm with a characteristic tridimensional architecture in which multicellular aggregates are surrounded by water channels forming a primitive circulatory system [1]. Bacterial biofilms produce about 90 percent of infections in humans and 65 percent of nosocomial infections according to the National Institutes of Health (NIH) and The Center for Disease Control (CDC) respectively [2]. Biofilms cause biofouling, water contamination, pipe plugging, product contamination, and equipment damage in industries; prostheses colonization, dental plaque, and disease in humans [3]. Biofilms also colonize burned tissue and open wounds.

Decontamination methods proven effective for free-living bacteria are typically ineffective against biofilms. Various mechanisms explain the resilience of biofilms to almost all forms of sterilization [4]. Therefore, new methods are required to solve the problem of biofilm eradication. Non-thermal atmospheric pressure plasmas is a methodology that shows promising results against biofilms and that has been researched for more than a decade [2,5–18]. Plasmas contain a mixture of reactive species, free radicals, and UV photons well-known for their decontamination potential against free microorganisms.

The effect plasma exerts on cell viability has been previously documented [5–10,13]. However, the effect on the biofilm matrix has received less attention and there is little evidence regarding the changes the matrix undergoes. Extracellular matrices are as diverse as biofilms and they contribute to the organization and the integrity of the community [19] and also to the resistance phenotype of the biofilm [20,21]. The contribution of each component of the EPS to the integrity of the matrix is not clearly understood at the molecular level. However, several functions of the EPS have been determined [22].

*Pseudomonas aeruginosa* is a biofilm-forming opportunistic pathogen that colonizes catheters, indwelling devices, and prostheses. The bacterium preys on victims with compromised immune systems such as patients on respirators, infects burned tissue, produces chronic rhinosinusitis [23], and colonizes lung tissue contributing to mortality in cystic fibrosis patients [24]. *P aeruginosa* co-colonizes lung tissue contributing to mortality in cystic fibrosis patients [24]. The eradication of *Pseudomonas* biofilms has been studied through a variety of approaches including surface modification [25,26], the use of antibiotics and biocides [27,28]; the use of chelators [29]; compounds such as furanone and *N*-acyl homoserine lactones [30,31] and also, the use of bacteriophages [32–34].

*P*. *aeruginosa* produces at least three matrix polysaccharides that differ in their chemical structure, function, and biosynthesis: Alginate, Psl, and Pel. Alginate is an *O*-acetylated linear β-(1→4)-linked polymer of D-mannuronic acid and its epimer L-guluronic acid [35]. Pel is a glucose-rich and cellulase-sensitive extracellular polysaccharide [36]. Lastly, Psl consists of a repeating pentasaccharide of D-mannose, D-glucose, and L-rhamnose [37]. *P*. *aeruginosa* PAO1 strain predominantly produces Psl associated to the matrix stability but it can also generate Pel [38]. Another component of the biofilm matrix is eDNA. Many contributions highlight the importance of eDNA in adhesion to surfaces and biofilm development [39]. It has been reported that eDNA functions as an intercellular connector in *P*. *aeruginosa* biofilms.

In most biofilms, less than 10% of the dry mass corresponds to microorganisms, whereas more than 90% accounts for the matrix [22]. In the tridimensional biofilm structure, the EPS function as a diffusion barrier for chemical compounds and antimicrobial agents and it is supposed to act as a complex defensive mechanism protecting bacteria from unfavorable environmental conditions [11]. The matrix also protects organisms against UV radiation, desiccation, oxidizing agents, some antibiotics and biocides, metallic cations, and host immune defenses, among others [22].

We preliminary reported the effects of a He plasma jet on the *P*. *aeruginosa* matrix using atomic force microscopy, AFM [2,8,40]. Vandervoort and Brelles-Mariño [2] studied the topography of plasma-treated *P*. *aeruginosa* biofilms and observed that under the conditions tested, the area corresponding to the matrix was reduced in the case of plasma-treated biofilms and that after 30 min of plasma treatment there were no structured areas that could putatively be assigned to the matrix. The researchers hypothesized that plasma could reduce the matrix areas by oxidation or peroxidation of the exopolysaccharides in the matrix by the reactive oxygen species present in the plasma. The reduction or eventual loss of the matrix would reduce the adhesiveness of the biofilm to the surface to which it is anchored and would lead to the disorganization or disintegration of the biofilm tridimensional structure. It was demonstrated that the adhesiveness of the matrix to the tip of an AFM cantilever varied throughout the biofilm surface and was reduced after plasma treatment. Similar effects were also reported by Ziuzina et al. [11,12] using scanning electron microscopy (SEM) and confocal laser scanning microscopy (CLSM). They demonstrated that an extended plasma treatment had a detrimental effect on the viability of *P*. *aeruginosa* both by disintegrating bacterial cells and also the biofilm matrix. Ziuzina et al. [11] reported a reduction in the thickness of plasma-treated biofilms compared to non-treated controls.

We have used a dielectric barrier discharge (DBD) plasma source operating in humidified air to treat *P*. *aeruginosa* biofilms and characterized the chemical composition of the effluent gas [18,41]. A similar DBD configuration has been proven effective against biofilms by other authors [11,12,42]. These authors, similarly to Vandervoort and Brelles-Mariño [2], observed a reduction of the biofilm thickness upon plasma treatment and concluded that although the exact mechanism of the plasma bactericidal mechanism is yet to be elucidated, the presence of RONS might be involved in the disintegration.

The aim of this work was to study the effect plasma exerts mostly on the *P*. *aeruginosa* biofilm matrix and to expand the existing knowledge about its effect on sessile cells in order to achieve a better understanding of the mechanism/s underlying plasma-mediated biofilm inactivation. In this paper we report a reduction in the amount of the biofilm matrix and the loss of its tridimensional structure together with major morphological changes in sessile cells at long exposure times. We report chemical and structural changes on the biofilm matrix (mostly on carbohydrates and eDNA) and cells (mostly on proteins and lipids) that are more profound with longer plasma exposure times. We also demonstrate the presence of lipid oxidation products thus confirming cell membrane lipid peroxidation as plasma exposure time increases. To our knowledge, this is the first report providing detailed evidence of the variety of chemical and structural changes that occur mostly on the biofilm matrix but also on sessile cells as a consequence of the plasma treatment. Based on our results, we propose an expanded model to explain plasma-mediated biofilm inactivation.

## Materials and methods

### Strain and culture medium

All the experiments were carried out with *Pseudomonas aeruginosa* PAO1 strain grown in AB minimal medium supplemented with 0.5% w/v glucose, unless otherwise stated [43]. Inocula were prepared from a plate in AB medium and grown in a shaker at 37°C and 180 rpm.

### Biofilm growth

For most of the experiments and unless otherwise stated, *P*. *aeruginosa* biofilms were grown on stainless-steel 316L 12.7 mm-diameter coupons in the CDC biofilm reactor (BioSurface Tech., MT) as described by Soler-Arango et al. [18]. Briefly, the biofilm reactor was inoculated with an overnight bacterial suspension grown in AB liquid broth to an optical density (OD) at 550 nm of 0.1 within the reactor vessel and operated under batch culture system at 37°C and 130 rpm for 24 h. Fresh medium was pumped thereafter through the biofilm reactor at a flow of 0.6 mL/min and spent medium was removed at the same rate. The flow was controlled with a LKB Bromma 2120 Varioperpex pump. The reactor operated as a chemostat with a fixed volume of 350 mL until constant OD for 24 h. After this time, coupons were aseptically removed from the reactor and unbound bacteria were removed by rinsing the coupons twice with sterile saline solution. Coupons were air-dried for 10 min and then placed biofilm side up into empty Petri dishes for treatment.

### Plasma generation and operating conditions

Atmospheric-pressure gas discharge plasma was produced using a DBD plasma reactor as described elsewhere [18]. Briefly, the plasma device has a coaxial geometry and a DBD configuration. The inner electrode consists of a 1 mm-diameter iron wire inside a 6 mm-external diameter glass capillary tube sealed at the tip. The outer electrode is a 25 mm-long aluminum tape attached to a 1 mm-thick acrylic 10 mm-diameter tube. The outer electrode is grounded. The ac power supply is a commercially available transformer for neon light (8 kV, 70 mA, and 50 Hz) connected to a variable autotransformer (Variac) to control the operating voltage amplitude.

The discharge operated with the power source at its maximum voltage of 8 kV (voltage amplitude at open circuit). Each electrode was connected to one of the two high-voltage transformer outputs and it was out of phase with the other one (16 kV between electrodes). The voltage was determined with a high voltage probe (Tektronix 1000 X /3.0 pf/100 MΩ) connected to the inner electrode. Current measurements were performed with a transformer (Bergoz CT-D5.0-B). These electrical waveforms were registered with a two-channel digitizing oscilloscope with a bandwidth of 60 MHz and a sampling rate of 1 Gs/s. As described in a previous contribution [18], air was used for plasma generation and the electric discharge took place at the interelectrode region and consisted of a series of short-lived filamentary micro-discharges. Typical waveforms of current and voltage applied to the inner electrode during the discharge showed the filamentary character of the discharge and also the existence of current peaks in the order of 10 mA with a negligible displacement current of ~ 0.01 mA superimposed [41].

In a previous contribution we characterized our plasma source by optical emission spectroscopy and detected the presence of excited N_2_ bands and N_2_^+^ ions and the formation of O_3_, H_2_O_2_ and NO_3_^-^ in the plasma afterglow [41].

For the biofilm experiments, the plasma applicator was mounted such that the discharge was 4 mm away from the coupon. The discharge was generated in moistened air at a flow of 1 L/min. Air was moistened by passing it through a humidifier filled with water and the air flow was determined using a commercial flow meter. The relative humidity of the air was 80 ± 5%. Humidity was determined with an analog hygrometer (Luft48HIG-DH) at the plasma applicator gaseous output. The temperature reaching the surface of the coupon was assessed with a thermocouple.

### Calcofluor White staining of the biofilm matrix

Calcofluor White staining (Calcofluor White M2R and Evans Blue 0.5 g/L, Sigma-Aldrich) was used to stain the biofilm matrix. Biofilms were grown and processed as described in section 2 and plasma-treated for 0, 3, 15, and 30 min. Then, 15 μL of a 1:1 mixture of Calcofluor White and 10% w/v potassium hydroxide were added to each coupon which was further incubated for 1 min in the dark. Stained biofilms were visualized with a Leica epifluorescence microscope using a 355/433 nm excitation/emission filter. Images were acquired with the Leica Application Suite (2.5.0 R1) with a 400X magnification and image analyses were performed with the ImageJ free software [44].

### Simultaneous staining of sessile cells and the biofilm matrix with SYTO9 and Calcofluor White

SYTO9 and Calcofluor White dyes were used to simultaneously stain biofilm cells and the biofilm matrix respectively. Biofilms were grown and processed as described above and plasma-treated for 0, 3, and 30 min. Coupons were covered with 15 μL of the SYTO9 stain and incubated in the dark for 20 min at room temperature. Excess dye was removed with sterile paper towels. Then, 15 μL of a 1:1 mixture of Calcofluor White and 10% w/v potassium hydroxide were added to the coupons that were further incubated in the dark for 1 min. Excess dye was removed as indicated before. Coupons were visualized with a Leica epifluorescence microscope using a 355/433 nm excitation/emission filter for Calcofluor White and a 480/500 nm filter for SYTO9. Images were acquired with the Leica Application Suite (2.5.0 R1) with a 400X magnification and image analyses were performed with the ImageJ free software.

### Digital treatment of the biofilm matrix and biofilm cell images

Once images were acquired as described above, the ImageJ software was used to determine the ratio between the areas of the biofilm matrix and the areas corresponding to biofilm cells. The digital treatment consisted of transforming the color images to a scale of grays and then binarizing them. Image binarization converts the grey scale in a black and white image (0 or 1) clearing the background and preserving the image details. In this case, we determined the area corresponding to black pixels. The ratio between the area of the binarized image of the biofilm matrix and the area of the binarized image of the biofilm cells was determined. The same procedure was carried out for plasma-treated and control biofilms.

### Scanning electron microscopy

Biofilms were grown on stainless steel coupons placed in a 24-well polypropylene microplate (Greiner CELLSTAR Sigma Aldrich). Each well was inoculated with 2 mL of a bacterial suspension in LB medium at a final OD_550nm_ of 0.1 and the microplate statically incubated for 24 h at 37°C. Then coupons were rinsed with 1 mL of sterile saline, air-dried, and plasma-treated for 0, 3, and 30 min. Samples were fixed in 2.5% (v/v) glutaraldehyde for 30 min and then rinsed in water to remove excess reagent. Dehydration was carried out by placing the coupons in a series of increasing concentration of cold ethanol solutions. The ethanol concentration in the dehydrating solution was 30, 50, 70, 90, and 95% v/v and each iteration lasted 20 min. Two additional increments at 100% ethanol for 20 min each were added to ensure complete ethanol saturation. All samples were critical-point dried in an EMITECH K850 dryer displacing ethanol with liquid CO_2_ and further evaporated at 31.1°C and 1072 psi. A ~15–20 nm gold sputter-coating was accomplished in a Balzers SCD 030 apparatus, and images were obtained using a SEM Philips 505 scanning electron microscope.

### Enzymatic degradation of matrix eDNA prior to plasma treatment

Biofilms were grown on stainless-steel coupons in the CDC biofilm reactor in AB medium under the conditions described in a previous section. Coupons were removed from the reactor, rinsed twice with sterile saline, air-dried for 10 min followed by the addition of 20 μL of bovine pancreas DNAse I (0.1 mg/mL) (Sigma-Aldrich); and a further incubation at 30°C for 60 min. The enzyme suspension was then removed with sterile paper towels and the coupons rinsed three times with 50 μL of sterile saline and air-dried for plasma treatment. Coupons were further treated with humidified air plasma for 0 (control), 3, 15, and 30 min, placed in a wet chamber, and incubated with 35 μL of sterile saline for 10 min. Two controls were included: one with enzyme but no plasma treatment, and one with neither enzyme nor plasma treatment. Biofilms were then scraped off and suspended in 1 mL of sterile saline, serially diluted, and 100 μL of each suspension was plated in duplicates on AB medium. Plates were incubated at 37°C and evaluated for colony-forming-unit (CFU) formation by counting the colonies. Survival curves (log_10_ CFU/mL versus plasma exposure time) were constructed and the decimal reduction time (D-value) calculated.

### Statistical analyses

The ratio between the area of the black pixels on the binary image of the biofilm matrix and the corresponding area of the biofilm cells was subjected to ANOVA analysis and t-Tukey test (S1 Table). The area of the FT-IR peaks corresponding to primary and secondary lipid oxidation products was also subjected to ANOVA analysis and t-Tukey test (S2 Table). The assumptions of normality and homocedasticity for the ANOVA were contrasted with the Kolmogorov-Smirmov test for normality, and the Levene test for the homogeneity of variances. ANOVA analysis and t-Tukey test (α = 0.05) to compare means were carried out using STATISTICA (StatSoft, 2011) software.

### Fourier Transform Infrared spectroscopy (FTIR)

#### Biofilm growth

For FT-IR analysis, biofilms were grown in sterile 24-wells polystyrene microplates. Each well was inoculated with an overnight culture of *P*. *aeruginosa* in AB liquid broth to an OD_550nm_ = 1 in a 2 mL final volume. The microplate was statically incubated for 24 h at 37°C. Spent medium was removed and wells were rinsed twice with sterile saline solution. Biofilms were air-dried for 10 min and further subjected to plasma treatment for 0, 3, and 30 min under sterile conditions.

#### Sample preparation and data acquisition

Biofilms grown on 24-wells polystyrene microplates were scrapped off and suspended in 100 μL of sterile saline after plasma treatment. The material recovered from two wells was pooled, deposited onto a ZnSe optical plate (13 mm diameter, Korth Kristalle GMBH, Germany) and air-dried for 1 h to obtain transparent films [45]. Three independent experiments or biological replicas were carried out. Each independent assay consisted of three technical replicates for each plasma exposure time (0, 3, and 30 min).

FT-IR absorption/transmission (A/T) spectra were acquired in the 4,000 to 600 cm^−1^ range with a FT-IR spectrometer (Bruker IFS 66, Bruker Optics) with 6 cm^-1^ spectral resolution and 64 scan co-additions [45,46]. To avoid interference with spectral water vapor bands, spectra were measured under a continuous purge of dried air.

#### Data pre-processing

All the acquired spectra were first subjected to a quality test (QT) using OPUS software [45]. This test included checking i) the absorbance in the amide I region (1,700–1,600 cm^−1^) with acceptable values between 0.20 and 1.20 absorbance units; ii) the noise signal (calculated from the first derivative between 2,100 and 2,000 cm^−1^) with values lower than 1.5×10^−4^; iii) the water vapor content (determined from the first derivatives between 1,847 and 1,837 cm^−1^) with values lower than 3×10^−4^. Only spectra that passed the QT were subjected to a pre-processing procedure. First, technical replicates were averaged getting three average spectra for each biological replicate and for each plasma exposure time (nine replicates in total). Then, second derivatives were calculated on these averaged spectra using the Savitzky-Golay algorithm with 9-point smoothing [45,47,48]. This procedure was carried out to increase the number of discriminative features inside the broad spectral bands, to help in the band assignment, and to minimize problems with baseline shifts. Finally, to avoid interference from biomass variations among the different samples, the second derivatives were vector-normalized in the full range. OPUS software (versions 4.2; and 7.0 Bruker Optics GmbH, Ettlingen, Germany) was used for spectral pre-processing.

#### Data analysis

Different cluster analyses (CA) were carried out in order to determine the heterogeneity and/or variability among different FT-IR spectra. For this purpose, the vector-normalized second-derivatives of the nine average spectra (see above) were used as input data. The spectral distance values were calculated at different wavenumber ranges as indicated in each case using the “scaling to first range” method, and clustering was carried out using Ward´s algorithm or average linkage. OPUS software was used for the analysis.

Semi-quantitative evaluation of the primary and secondary lipid oxidation products was determined calculating the band areas at the two main peaks: 1,745 cm^−1^ (1,780–1,735 cm^−1^ region) and 1,715 cm^−1^ (1,730–1,710 cm^−1^ region) in the three vector-normalized average spectra obtained for each plasma exposure time. As these two bands overlap under the shoulder of the amide I band at 1,743 cm^-1^, the areas were calculated after a deconvolution and fitting procedure. The frequencies and the half-width of each peak used for a least-squares iterative curve-fitting procedure were those obtained from the second derivative of the original spectra [49]. The areas of the bands were calculated by integration of the corresponding fitted bands. Data preprocessing, curve fitting, and peak-area calculation were carried out with the OPUS software.

To determine the heterogeneity among the FT-IR spectra, the variability in the biological replicates of each plasma exposure time was calculated as previously described [45]. Briefly, the spectral variance was determined as the average ±2 standard deviations of the so-called spectral distance (D). D corresponds to a dissimilarity measure, equal to (1-*r*) × 1000, being *r* the Pearson's correlation coefficient [47]. The spectral distances, calculated in the whole spectral range (3,000–2,800 cm^−1^; 1,800–1,550 cm^−1^; 1,500–1,250 cm^−1^ and 1,200–900 cm^−1^) were 3.40 ± 1.91, 3.53 ± 0.93 and 2.51 ± 2.17 for *P*. *aeruginosa* biofilms exposed to plasma for 0 (control), 3, and 30 min respectively (see S1 Fig). Spectral distances higher than the cut-off number of D = 6 (marked with dotted lines in dendrograms) indicate that plasma produced significant changes in the structure or chemical composition of the samples analyzed.

## Results and discussion

### The amount of biofilm matrix is reduced after plasma treatment

Calcofluor White is a non-specific fluorochrome that binds to a variety of polysaccharides and has been used to stain the biofilm extracellular matrix. The dye strongly interacts with glycosydic bonds mainly β-(1→4) and β-(1→3) [50].

**Fig 1:**
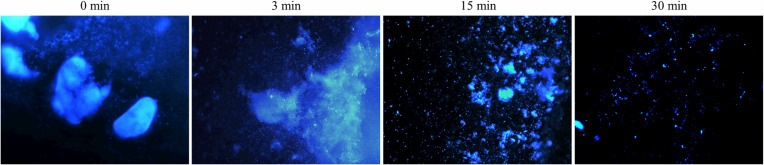
Plasma-treated *P*. *aeruginosa* biofilms stained with Calcofluor White. Biofilms were treated with plasma for 0, 3, and 30 min prior to staining. Images were acquired with a 355/433 nm excitation/emission filter at 400 X and processed with the Leica Application Suite software.

Fig 1 shows microscopy images of *P*. *aeruginosa* biofilm matrices stained with Calcofluor White. Control biofilms with no plasma exposure and biofilms treated with plasma for 3 min show big dense aggregates of matrix. Biofilms treated with plasma for 15 and 30 min depict a more disperse and disorganized matrix.

**Fig 2:**
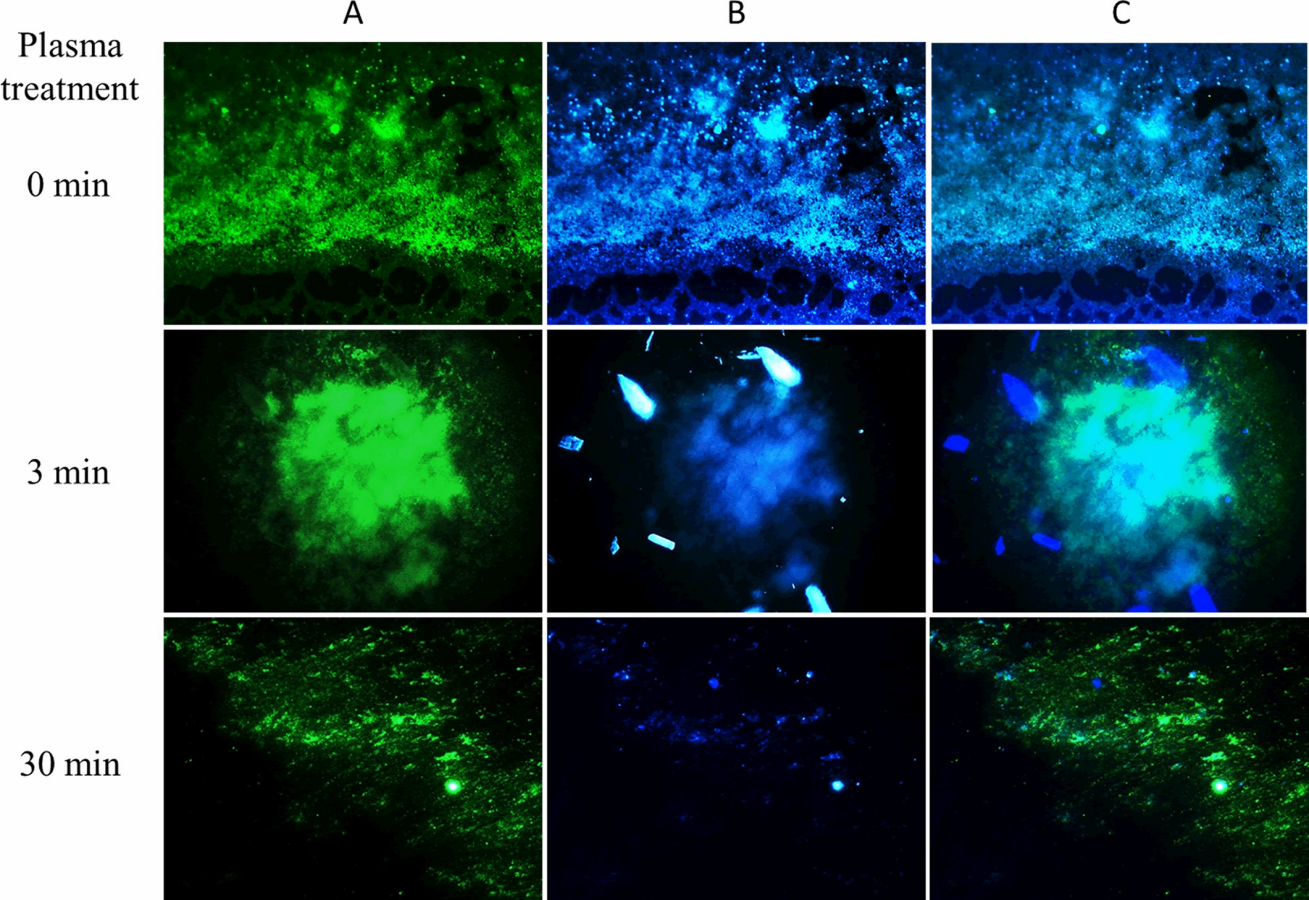
Plasma-treated *P*. *aeruginosa* biofilms stained with SYTO9 and Calcofluor White. Biofilms were treated with plasma for 0, 3, and 30 min prior to staining. Column A depicts images corresponding to SYTO9 staining and visualized with the 480/500 nm excitation/emission filter. Column B shows images of the same samples subjected to Calcofluor White staining and obtained with a 355/433 nm excitation/emission filter. Column C depicts superimposed images from the previous two panels. 400 x images were acquired with the Leica Application Suite software.

Fig 2 shows the simultaneous staining of the biofilm with Calcofluor White and SYTO9. The green fluorescent nucleic acid stain SYTO9 stains live and dead bacterial cells while Calcofluor White stains predominantly matrix. Images show numerous cells covered and surrounded by matrix in the control biofilm (0 min plasma treatment). There are still abundant cells and matrix upon a 3-min plasma treatment whereas the amount of polysaccharidic matrix is drastically reduced after treating the biofilm with plasma for 30 min.

**Fig 3:**
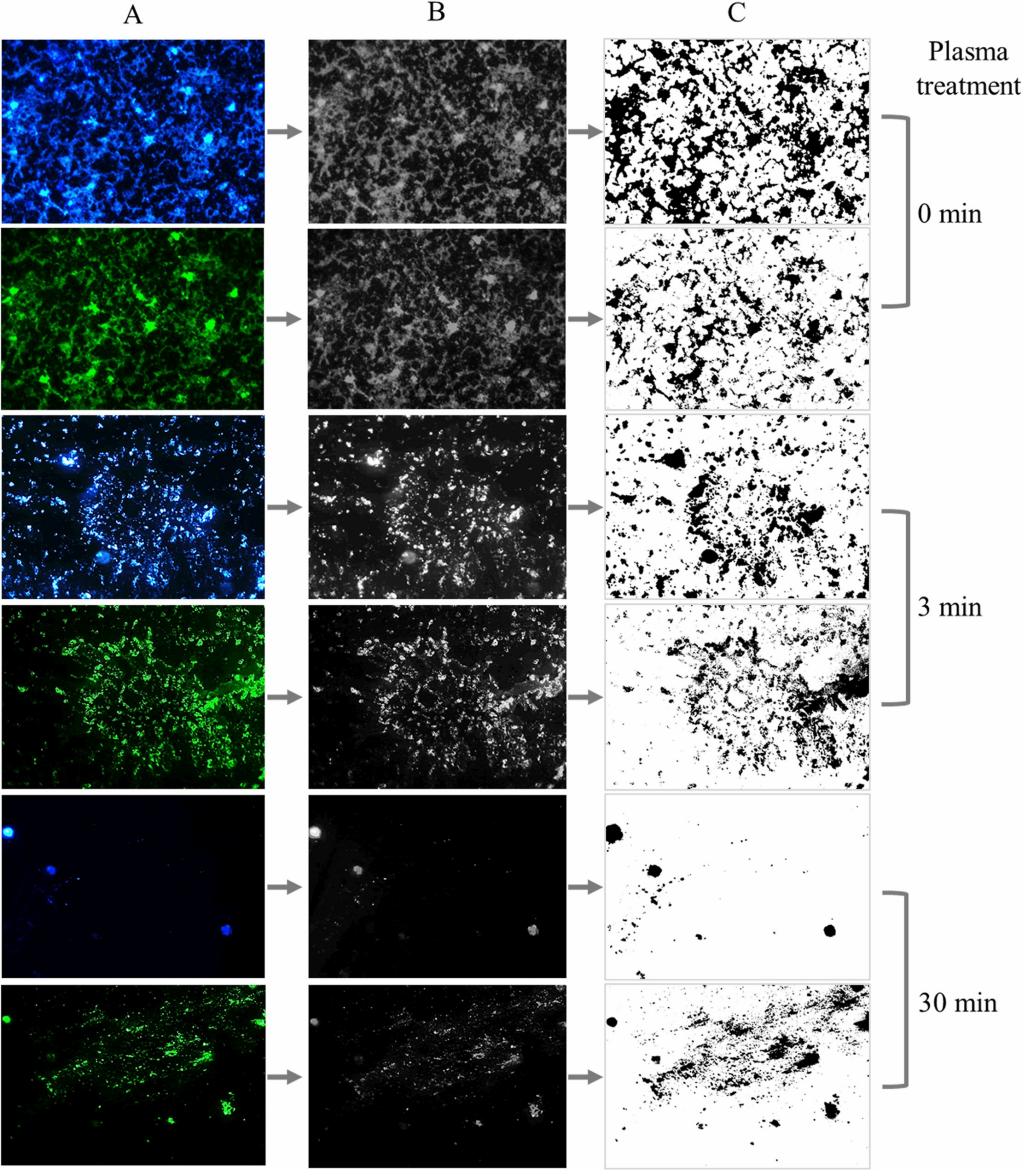
Image binarization of plasma-treated *P*. *aeruginosa* biofilms stained with SYTO9 and Calcofluor White. Biofilms were treated with plasma for 0, 3, and 30 min prior to fluorescent staining with Calcofluor White for the biofilm matrix (blue) and SYTO9 for the bacterial cells (green). 400 X images were acquired with the Leica Application Suite software. Column B corresponds to image digitalization to black and white and column C shows the further binarization with ImageJ software (right panel).

In order to quantify changes in the biofilm matrix and sessile cells after plasma treatment, double-stained images were binarized with the ImageJ software. Fig 3 shows biofilms treated with plasma for 0, 3, and 30 min and stained either with Calcofluor White or SYTO 9. Images were first digitally converted into a scale of grays and then into black and white pixels for further analysis.

Table 1 shows the ratio between the area of the binarized image of the biofilm matrix and the area of the binarized image of the biofilm cells. For biofilms not exposed to plasma, the ratio is ˃ 1.5 indicating a higher amount of matrix compared to sessile cells. The ratio decreases upon plasma treatment and drops to figures closer to 0.5 for biofilms exposed to plasma for 30 min.

**Table 1:** Ratio between the biofilm matrix area and the sessile cells area obtained from binary images of plasma-treated biofilms.

Plasma exposure time (min)	matrix area/cells area
0	1.75 ± 0.12 ^a^
3	1.05 ± 0.13 ^b^
30	0.46 ± 0.10 ^c^

Ratio between the black pixel area in the binary image of the biofilm matrix stained with Calcofluor White and the black pixel area in the binary image of the biofilm cells stained with SYTO9. Biofilms were treated with plasma for 0, 3, and 30 min prior to staining. Images were binarized as indicated in Fig 3. Means are the average of the ratio of areas from 40 pictures acquired in 4 independent experiments. Different letters correspond to significantly different means (p<0.001).

Most of the studies on plasma-mediated biofilm eradication focus on the effect of plasma on bacterial sessile cells and very few refer to the damage inflicted to the biofilm matrix. We previously reported the effects of plasma on the *P*. *aeruginosa* matrix using atomic force microscopy, AFM [2,8,40]. Vandervoort and Brelles-Mariño [2] studied the topography of plasma-treated *P*. *aeruginosa* biofilms and observed that the area corresponding to the matrix was reduced in the case of plasma-treated biofilms compared to control ones and that after 30 min of plasma treatment there were no areas that could putatively be assigned to the matrix. The researchers suggested that plasma could reduce the matrix areas possibly by oxidation or peroxidation of the exopolysaccharides by the reactive oxygen species present in the plasma. The reduction or eventual loss of the matrix would reduce the adhesiveness of the biofilm to the surface to which it is anchored and would lead to the disorganization or disintegration of the biofilm tridimensional structure. It was demonstrated that the adhesiveness of the matrix to the tip of an AFM cantilever varied throughout the biofilm surface and was reduced after plasma treatment. The effect of plasma on the biofilm matrix was also reported by Ziuzina et al. [11,12] using scanning electron microscopy (SEM) and confocal laser scanning microscopy (CLSM). They demonstrated that an extended plasma treatment had a detrimental effect on the viability of *P*. *aeruginosa* both by disintegrating bacterial cells and the biofilm matrix.

Our results show a reduction to an almost complete removal of the biofilm matrix after plasma exposure. Taking into account that the biofilm cells undergo morphological and physiological changes upon plasma treatment, it is evident that biofilm eradication is not only due to cell death but also to matrix degradation/disintegration. Reactive species and UV radiation in the plasma might chemically modify and degrade the matrix leaving the cells more vulnerable to the treatment.

### The structure of the biofilm matrix is altered after plasma treatment

**Fig 4:**
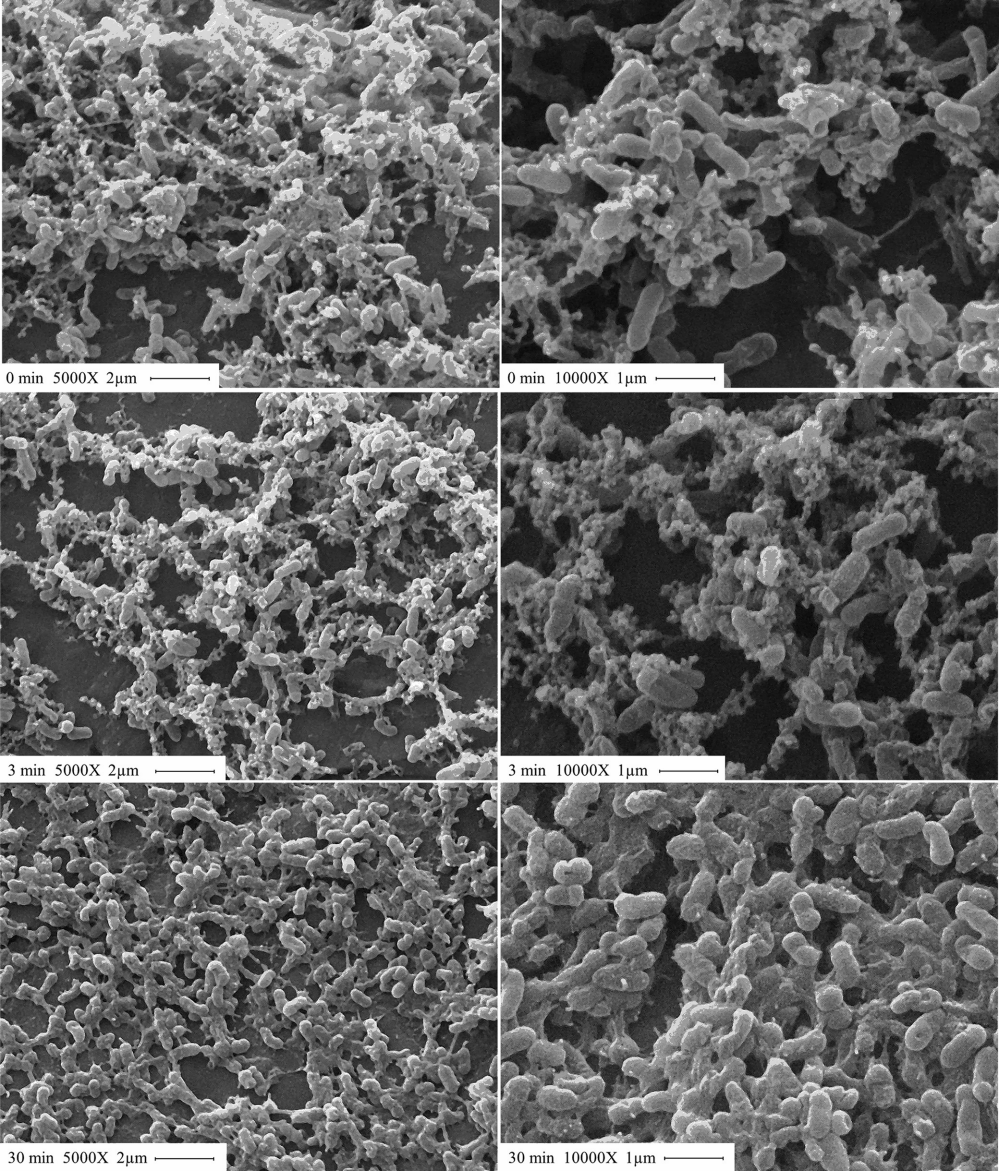
SEM images of plasma-treated *P*. *aeruginosa* biofilms. Biofilms were treated with plasma for 0, 3, and 30 min (from top to bottom). Magnification: 5000X and 10000X for images on the left and right panels, respectively.

Fig 4 shows the SEM images of *P*. *aeruginosa* biofilms treated with plasma for 0, 3, and 30 min. Control biofilms depict a tridimensional structure where 1 μm long, intact rods are surrounded by matrix and interwoven with matrix fibers. The images corresponding to biofilms treated with plasma for 3 min show some cells of irregular shape but there is no significant damage either to the matrix or to the cell morphology. After a 30-min exposure to plasma, images depict a flat, disorganized biofilm with distorted and broken cells and matrix disintegration. Several authors have used SEM to assess the effect of plasma on *P*. *aeruginosa* sessile cells [14,15,51] whereas very few have used it to evaluate the effect on the biofilm matrix. Ziuzina and colleagues [11,12] used SEM to visualize the effect of a DBD plasma source on *E*. *coli* and *P*. *aeruginosa* biofilms formed on polycarbonate membranes. For *P*. *aeruginosa* biofilms and, in agreement with our results, these authors observed intact cells surrounded by matrix and interwoven with matrix fibers in the biofilm control and deformed cells and matrix disintegration after 5 min of plasma treatment.

### eDNA protects the biofilm against plasma treatment

Many contributions highlight the importance of eDNA in adhesion to surfaces and biofilm development [39]. Biofilm-forming strains of *P*. *aeruginosa* have been shown to produce extracellular eDNA that might function as a cell-to-cell interconnecting compound within the biofilm matrix [39,52]. Allesen-Holm et al. [52] treated *P*. *aeruginosa* biofilms of different ages with DNase and found that the enzyme dissolved young biofilms while the established ones were marginally affected by the treatment. These results suggest that young biofilm cells are held together by eDNA whereas there are other compounds that play this role in older biofilms.

**Fig 5:**
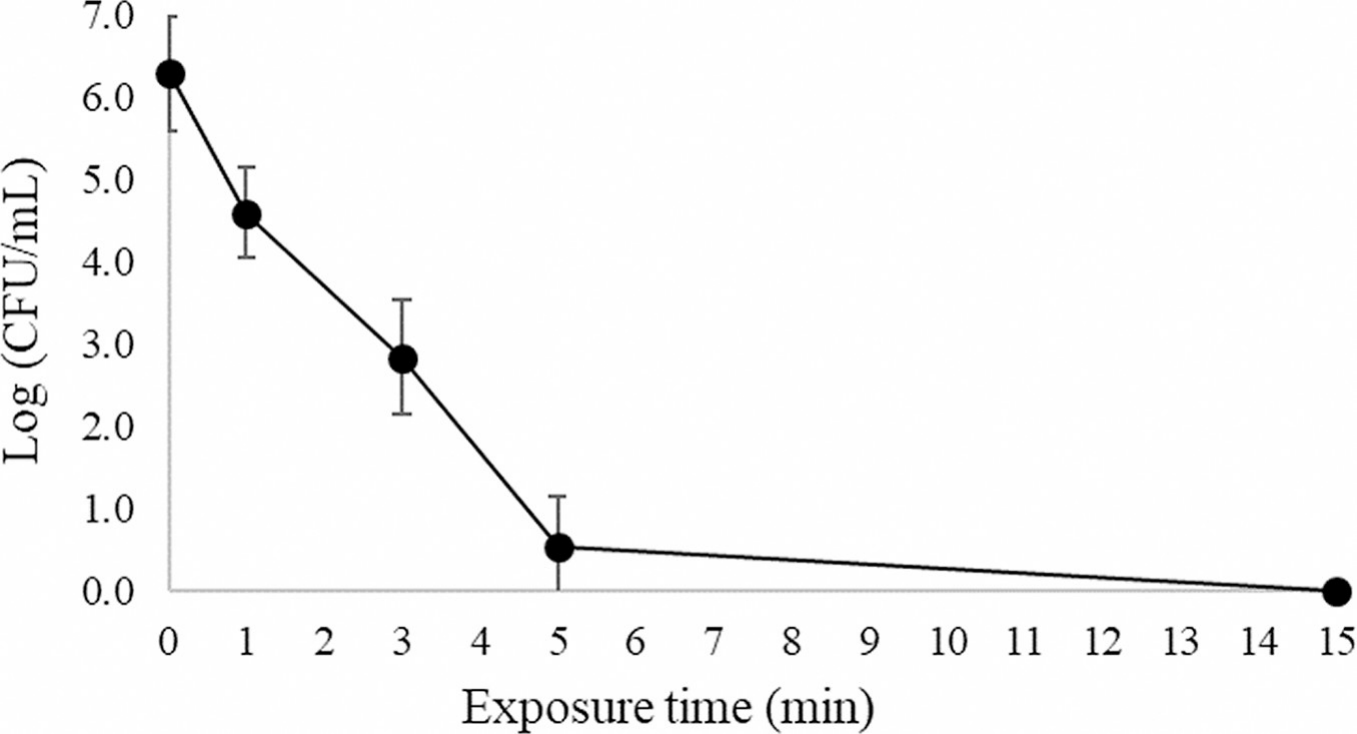
Bacterial survival curve. *P*. *aeruginosa* biofilms grown in continuous culture on stainless-steel coupons in AB synthetic medium were treated with DNase and exposed to plasma generated in moistened air. Results are the average of four independent experiments. Each experiment was performed in duplicates. Error bars represent the standard error of the mean.

In a previous contribution we demonstrated the presence of abundant extracellular DNA (eDNA) on the *P*. *aeruginosa* biofilm matrix [18]. To determine whether eDNA helps protecting the biofilm matrix from plasma, eDNA was enzymatically degraded with DNase before plasma treatment. Fig 5 shows the survival curve of a DNase-treated biofilm subjected to plasma for different exposure times. The curve shows double-slope kinetics with a rapid decline in the number of CFU/mL for the first five min followed by a slow decline for longer exposure times. A decrease of 3.4 log units in the number of CFU/mL was obtained after three min of exposure to plasma and biofilms were completely eradicated with a decrease of 6.3 log units (˃99.999% killing efficacy) in the number of CFU/mL after a 15-min plasma treatment.

Two controls were included in this experiment: a non-treated biofilm and a biofilm subjected to DNase but with no plasma treatment. The biofilm subjected to DNase treatment showed a modest decrease in the number of CFU/mL of 13.4% compared to the control with no treatment, probably due to the detachment of cells during the rinses after the enzymatic treatment. This result suggests that although eDNA may not play an instrumental role in the maintenance of the biofilm structure, it functions as a link among the outer biofilm cells.

Plasma decontamination efficiency can be assessed by determining the decimal reduction time (D value), time required to reduce the microorganism concentration by 90%. This parameter has been originally defined for thermal killing of microorganisms by autoclaving. The first portion of the survival curve has a D value (D_1_) of 0.91 ± 0.07 min for short exposure times. For biofilms not subjected to DNase treatment, we previously reported a similar double-slope inactivation kinetics with a D value (D_1_) of 1.14 ± 0.28 min [18]. These two D-values were significantly different (p<0.05). These results show that eDNA plays a role in the protection of the biofilm since its removal results in a biofilm that is faster and therefore easier to eradicate with plasma than the biofilm with an eDNA-containing matrix. Abramzon et al [5] suggested that the double-slope kinetics could be explained by the death of the biofilm upper layers of microorganisms, readily available and more exposed to plasma. After this rapid initial inactivation, plasma has to penetrate layers of cell debris and dead cells before reaching the inner portion of the biofilm. Based on our results, it is clear that after eDNA removal, cells are more exposed to plasma and its reactive species can reach easier the inner portion of the biofilm leading to a more effective biofilm eradication.

### The chemistry and structure of the biofilm macromolecules are altered after plasma treatment

**Fig 6:**
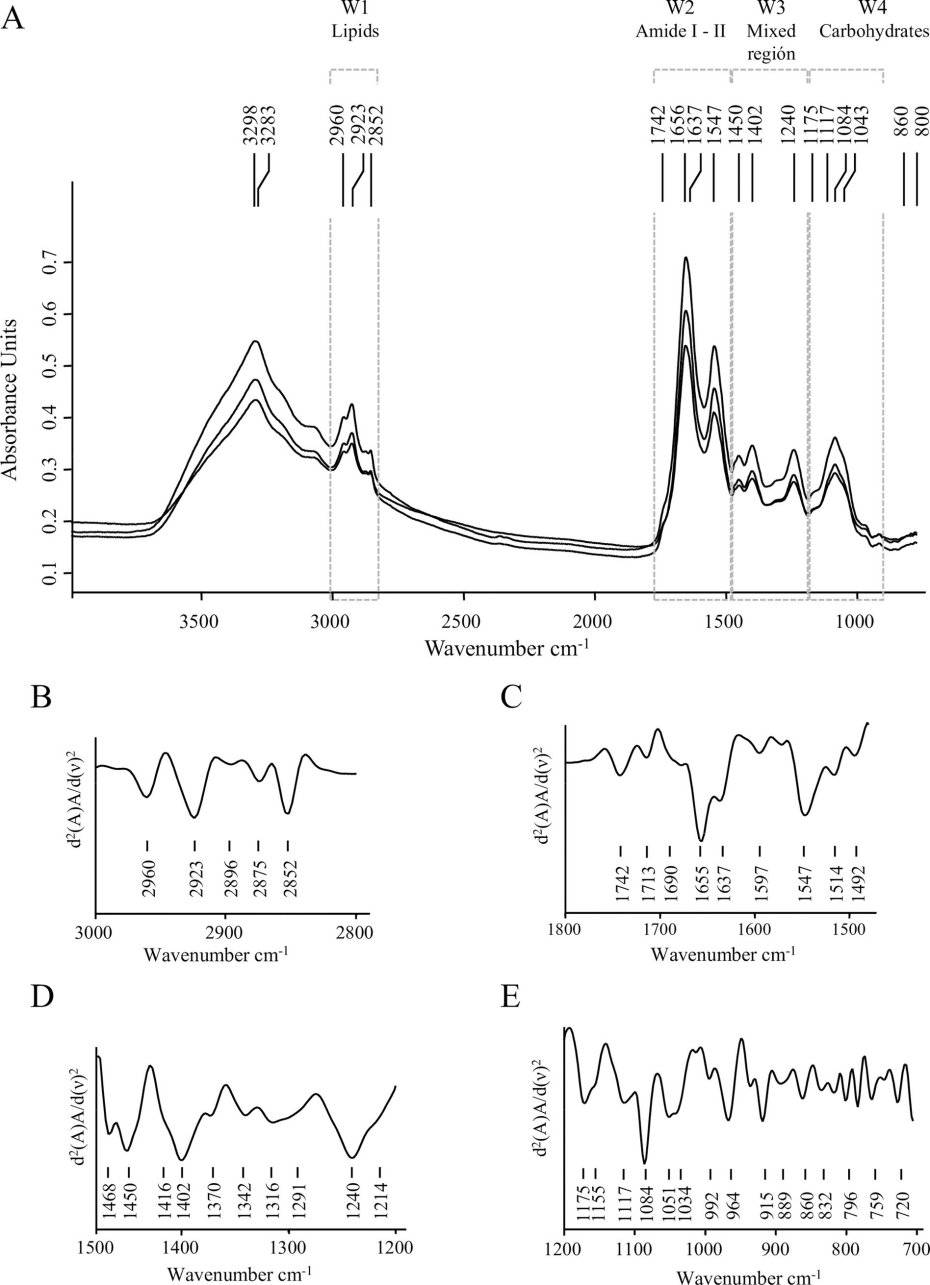
A/T FT-IR absorption spectra of *P*. *aeruginosa biofilms*. (A) Three average spectra obtained from three independent experiments corresponding to *P*. *aeruginosa* biofilms with no plasma treatment (control samples). Spectral windows (W1 to W4) associated to functional groups in biomolecules are indicated. (B-D) second derivatives at the different spectral windows: (B) W1 spectral windows associated to lipids, (3,000–2,800 cm^-1^), (C) W2 region assigned to protein absorptions (1,800–1,500 cm^-1^), (D) W3 the mixed region (1,500–1,200 cm^-1^), and (E) W4 assigned to carbohydrates absorption bands (1,200–900 cm^-1^).

Fourier transform infrared spectroscopy (FT-IR) provides information on the overall biochemical and structural composition of the material under study. In this work, A/T FT-IR dry film technology was used to evaluate the whole cell and matrix composition of *P*. *aeruginosa* biofilms and the chemical and structural modifications macromolecules go through when biofilms are treated with plasma for different exposure times. Fig 6A depicts the three average spectra obtained from three independent experiments corresponding to *P*. *aeruginosa* non-treated biofilms (control samples). The second derivative of one of those average spectra at the main spectral windows is shown in Fig 6B–6D. The FT-IR absorption spectra show the overall spectral features and the particular markers previously reported for biofilms produced by *P*. *aeruginosa* strains and other Gram-negative biofilms [53–55]. The well-defined spectral windows associated to functional groups in biomolecules are indicated as follows: W1 (3,000–2,800 cm^−1^), the spectral region assigned to C-H vibrational modes in–CH_3_ and >CH_2_ asymmetric and symmetric stretching modes of fatty acids and lipids. W2 (1,780–1,480 cm^−1^), mainly indicative of biomass content, includes amide I (1,780–1,600 cm^−1^) and amide II (1,600–1,480 cm^−1^) regions. Amide I contains the >C = O stretching vibrations in amide bonds of proteins, in esters mainly from phospholipids, carboxylic acids, and lipid oxides; and the >C-OO• functional group in lipid peroxides. This region is also sensitive to changes in protein secondary structure. Amide II region is assigned to C-N stretching and N-H bending in protein and peptides. W3 (1,500–1,200 cm^−1^) the so-called “mixed region”, contains the spectral bands at 1,450, 1,402, and 1,240 cm^−1^ most probably resulting from a weak band due to C–H bending vibrations of CH_2_, >C–O bending from carboxylic acids, and >P = O symmetric stretching corresponding to fatty acid, proteins, and phosphorus-containing carbohydrates, respectively. Finally, W4 (1,200–900 cm^−1^) the “carbohydrates window” contains the spectral bands of cell carbohydrates and other matrix components [45,56] (Table 2).

**Table 2:** Spectral windows associated to functional groups in biomolecules and band assignment.

Windows, spectral regions	Wavenumber (cm^-1^)	Band assignation^1^		References
WA 3,300–3,100	3,298 3,283	N-H and O-H str. vibrations in polysaccharides and proteins		[45,46,59]
W1 Lipids 3,000–2,800	2,960	C-H asym. str. of -CH_3_ in fatty acid chains		[45,46]
	2,923	C-H asym. str. of >CH_2_ in fatty acid chains		
	2,875	C-H sym. str. of -CH_3_ in fatty acids		
	2,852	C-H sym. str. of >CH_2_ in fatty acids		[45,46,59–62]
W2 Amide I and II 1,780–1,480	1,740–1,745	>C = O str. in carboxylate ions, esters in lipids, fatty acids peroxides		[45,46,55,63,64]
	1,720–1,715	>C = O str. in COOH group of fatty acids, lipid oxides		
	Amide I 1,650	>C = O str. and C–N bending in amide groups of proteins and peptides. Sensitive to protein conformation	1,695–1,675 β-antiparallel	[49,59,65–67]
			1,670–1,666 β-turns	
			1,660–1,650 α-helix	
			1,635–1,625 β-sheets	
	Amide II 1,540	N–H bending, C–N str. of proteins and peptides		
W3 Mixed region 1500–1200	1,450	C-H bend in CH_2_ and CH_3_		[45,46]
	1,402	>C = O sym. str. for deprotonated COO^-^ group and C-O bending from COO^-^		[45, 55, 60–62]
	1,240	P = O asym. str. of PO_2_¯ in phosphodiesters of DNA/RNA		[46, 54, 58, 62]
W4 Carbohydrates1200-900	1,175 1,117 1,084 1,043	C–OH str., C–O–C and C–O ring vibrations in polysaccharides and C-P-O str.		[45, 54–56,62,68]
Fingerprinting 900–700	860	α- glycosidic linkages		[69]
	800	β- glycosidic linkages		[55,69]
	720	C-H rocking of CH_2_		[45]

1 asym: asymmetric; sym: symmetric; str: stretching; ben: bending.

The four typical bands due to C–OH stretching modes, C–O–C, and C–O ring vibrations in polysaccharides were detected (Table 2). It is important to note that under our experimental conditions, the reported bands due to alginate within the biofilm matrix such as the shoulder on the amide I band at 1,615 cm^−1^ (asymmetric stretching of the carboxylate ion), the peaks at 1,250 cm^−1^ (C-O-C of the esters in alginate), and 1,060 cm^−1^ (C-OH stretching of alcohols) were not observed [54]. These results are in agreement with those of Wozniak et al. [57] who reported that alginate is not a significant component of the extracellular matrix of *P*. *aeruginosa* PAO1 biofilms. Besides, bands present at W1 and W4 might be indicating that, under our experimental conditions, the biofilm matrix contains mainly Psl composed of D-mannose, D-glucose, and the desoxyhexoses l-rhamnose [37]. DNA absorption bands were also detected at 1,084 cm^−1^, assigned to the vibration of C-O-C and C-O-P in polysaccharides but also to PO_2_¯ groups in nucleic acids; and at 1,240 cm^−1^, mostly from the vibration intensities of the phosphodiester PO_2_¯ groups in the DNA/RNA backbone structure [54,58].

**Fig 7:**
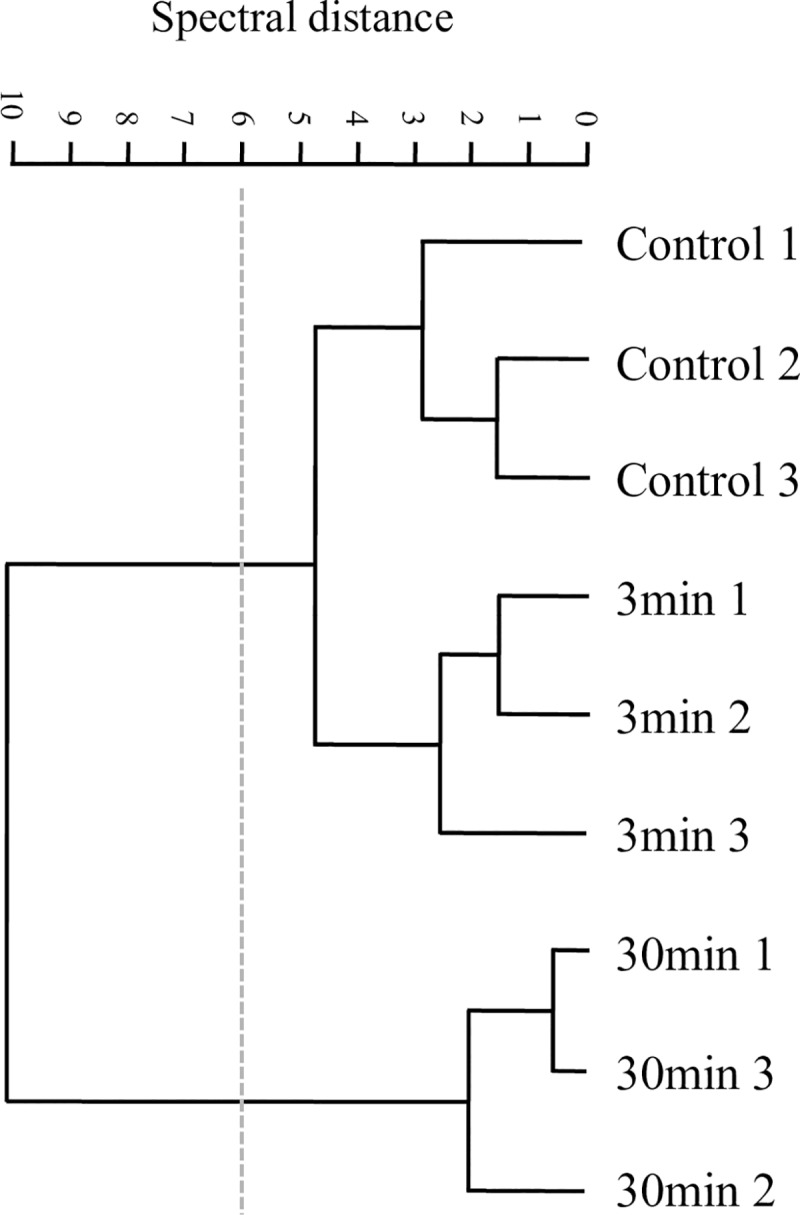
Plasma effect on whole *P*. *aeruginosa* biofilms. Cluster analysis obtained with 2^nd^ derivatives as input data using scaling to first range for spectral distance calculation in the entire IR spectrum: (3,000–2,800 cm^-1^), (1,800–1,550 cm^-1^), (1,500–1,250 cm^-1^), (1,200–900 cm^-1^). Dendrogram was constructed using Ward´s algorithm. The cut-off value of D = 6 is indicated with the dotted line.

As a first approach, we analyzed the effects of plasma on *P*. *aeruginosa* biofilms comparing the features of the second derivatives of spectra obtained after different plasma exposure times in the whole IR range. This first FT-IR analysis confirmed that cells and matrix macromolecules and structures were modified, being the impact of this effect more evident after 30 min of plasma treatment. A hierarchical cluster analysis using second derivatives as input data showed that those changes occurred in sessile biomass (W2 and W3), cell membranes (mainly covered by W1), matrix-included carbohydrates, and DNA (W1 to W4). It is evident that profound changes took place when plasma treatment was extended from 3 to 30 min (Fig 7).

**Fig 8:**
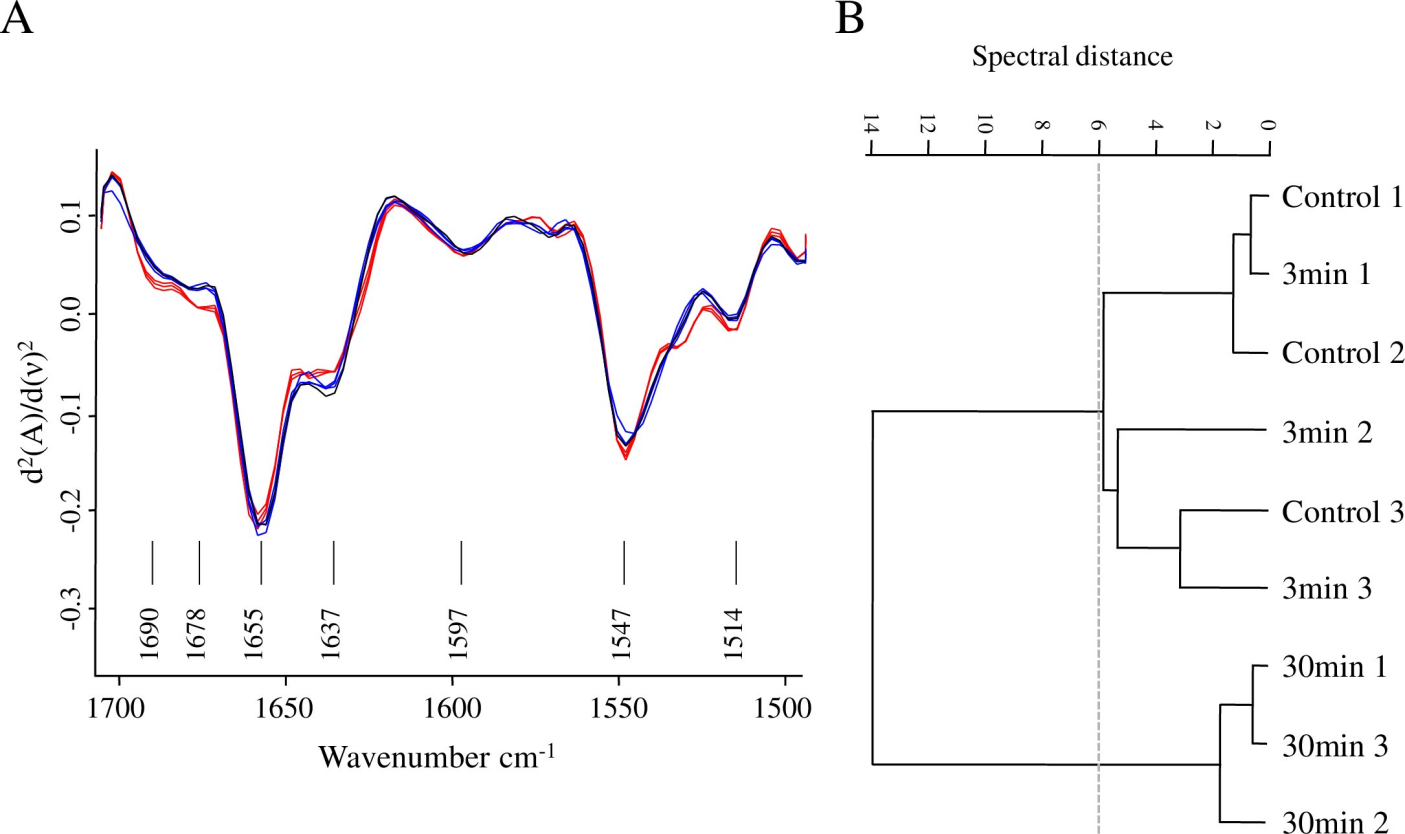
Plasma effect on protein conformation. (A) Vector-normalized second-derivatives of the average spectra of plasma-treated biofilms (blue, 3 min; red, 30 min; and black, control) in the amide I and II regions. (B) Cluster analysis obtained with 2^nd^ derivatives as input data using scaling to first range for spectral distance calculation in the spectral ranges: (1,669–1,697 cm^-1^), (1,633–1,661 cm^-1^). Dendrogram was constructed with Ward´s algorithm. The cut-off value of D = 6 is indicated with the dotted line.

In order to carry out a more in depth analysis of the effect of plasma on sessile cells, we studied the spectral features of amide I band (~1,650 cm^−1^), which gives information of protein conformation; and amide II band (~1,540 cm^−1^) which, together with amide I are considered biomass markers [45,70]. Through amide I band it is possible to evaluate protein and biomass alterations by changes in the contribution of α-helix, parallel and anti-parallel β-sheet, β-turns, and unordered conformations (Table 2). Although there are some difficulties and limitations in discriminating the contribution of each conformation within the overall secondary structure of proteins, the use of the second-derivative technique allows overcoming some of these limitations [66,71]. When the vector-normalized second derivative of the average spectra for the three conditions studied (plasma treatment for 0, 3, and 30 min) were compared, significant differences among contributions of the peaks under amide I and amide II bands were found (Fig 8). The conformational distribution of proteins in *P*. *aeruginosa* biofilms seems to be mainly a combination of α-helices and β-sheets conformation observed at approximately 1,655 and 1,630 cm^−1^, respectively. Interestingly, as plasma treatment increased from 3 to 30 min, a decrease in the intensity of the peak assigned to β–sheet conformation together with an increase in the intensity of the bands assigned to β-turns and antiparallel β-sheets were observed (Fig 8A, Table 2). In antiparallel β-sheet structures, the chain packing has a lower total energy and peptide dipoles alignments are more favorable than in parallel β-sheets [72]. Therefore, the β-parallel sheet motifs in the biofilm proteins seem to change under plasma treatment to joints of β-turns and β-antiparallel sheet producing the typical super-secondary stable pattern which is regularly repeated [73]. In addition to protein structure modifications, plasma effect on sessile biomass could also be observed through amide II changes. The dendrogram built with the vector-normalized second derivative spectra in the amide I and II regions (Fig 8B) provides evidence that the biofilm exposure to plasma produces significant modifications in the biomass content and denaturation or aggregation of proteins mostly after 30 min of treatment.

Nevertheless, one of the key factors most probably contributing to biomass damage in the biofilm is the injury produced on lipid cell membranes by plasma reactive species. Lipids are particularly susceptible to free radical activity leading to oxidative degradation or peroxidation. HOO•, and O_2_•^-^ radicals, singlet oxygen, and O_3_ can initiate peroxidation of unsaturated fatty acids and launch a peroxidation chain reaction. The lipid peroxidation chain reaction begins after hydrogen removal from an unsaturated fatty acid to form a lipid radical that reacts with molecular oxygen to form a lipid peroxy radical (ROO'). This radical will attack another unsaturated fatty acid to form a fatty acid hydroperoxide (ROOH) perpetuating the initial reaction. These primary oxidation products are further broken down into secondary products with shorter hydrocarbon chains. The peroxidation of lipids thus generates products which are shorter than the initial fatty acid [74]. The chemical products of lipid peroxidation are therefore, lipid peroxides (LOOH) and lipid oxidation products (LOPs). The latter are non-radical products, mostly aldehydes and ketones such as L = O lipid oxides (LO) and hydroxides (LOH) [63,74,75].

FT-IR spectroscopy allows the monitoring of bands whose changes indicate the formation of primary and secondary oxidation products [63,64,75–77]. At 1,740–45 cm^−1^, there is a very important band associated with stretching vibrations of the carbonyl group >C = O, particularly in ester bonds between fatty acids and glycerol within the lipid molecule. This band is also associated with the formation of primary oxidation products of fatty acid chains in lipid peroxidation (LOO• and LOOH) (Table 2). On the other hand, the absorbance of the aldehyde functional group in LOPs overlaps with the one corresponding to >C = O in carboxylates or acids at 1,750 cm-1 in the amide I band [63,64,76]. Therefore, the evaluation of the >C = O stretching band intensity at 1,743 cm^−1^ and 1,715 cm^-1^ in vector-normalized spectra corresponding to biofilms exposed to plasma for 0, 3, and 30 min, can be used as cell membrane lipid peroxidation markers. Our results did not show the expected increase of the band intensity at 1,745 cm^-1^ due to primary oxidation products (Table 3). However, the increase of the final oxidation products directly depends on the generation of the primary oxidation products [77]. In our case, we assume that while the >C = O stretching band intensity at 1,743 cm^-1^ might increase due to the accumulation of LOO• products, a significant decrease occurred due to cleavage of the ester link probably as a result of matrix degradation/disintegration. Nevertheless, after 30 min of plasma exposure, the concentration of radical species LOO• and L• is high enough for the species to collide generating stable species and the secondary oxidation products detected at 1,715 cm^-1^ (Table 3) [76,77]. Based on the thorough analysis of the modification of >C = O band features described above, we can conclude that there is a clear significant damage to cell proteins and peroxidation of cell membrane lipids as plasma exposure time increases.

**Table 3:** Semi-quantitative analysis of primary lipid oxidation products and final oxidation products.

Plasma exposure time (min)	Primary lipid oxidation products and >C = O in ester bonds	Final oxidation products and >C = O in carboxyl acids
0	1.27 ± 0.06 ^a^	0.67 ± 0.02 ^c^
3	1.16 ± 0.04 ^a^	0.64 ± 0.06 ^c^
30	0.79 ± 0. 05 ^b^	0.76 ± 0.01 ^d^

Primary and secondary lipid oxidation products were measured by spectral band areas at 1,740–45 cm^-1^ and 1,715–20 cm^-1^, respectively. Spectral band area values were obtained from the average spectra calculated at each plasma exposure time (0, 3, and 30 min). Results are the average of three independent experiments. Each experiment was performed in duplicates. Different letters indicate significant differences at α = 0.05.

**Fig 9:**
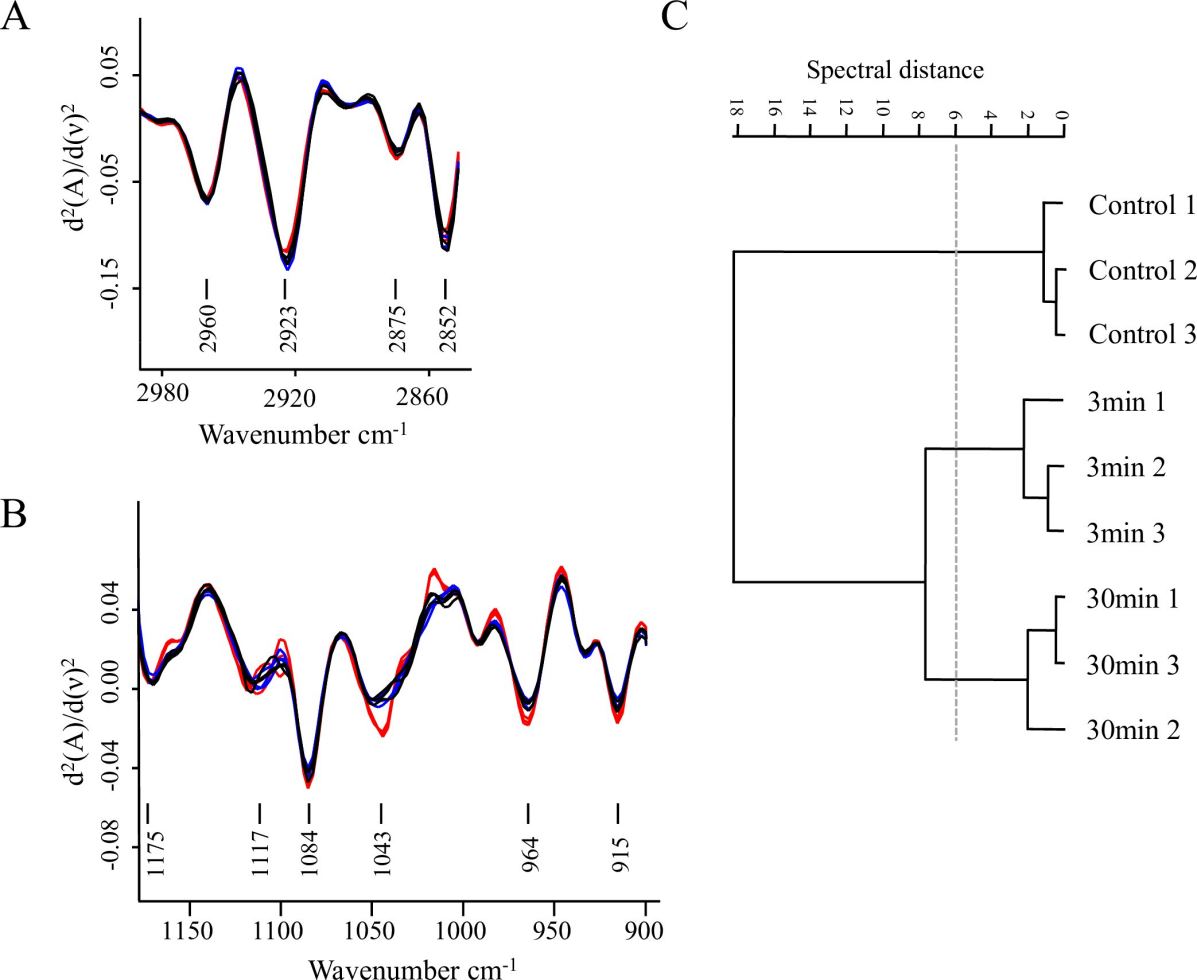
Plasma effect on biofilm carbohydrates. (A, B) Vector-normalized second-derivatives of the average spectra in the C-H stretching vibration region (W1 3,000–2,800 cm^-1^) (A), and in the carbohydrate region (W4 1,200–900 cm^-1^) (B). Black, blue, and red lines correspond to biofilms treated with plasma for 0, 3, and 30 min, respectively. (C) Cluster analysis obtained with 2^nd^ derivatives as input data using scaling to first range for spectral distance calculation in the spectral ranges: 2,880–2,870 cm^-1^ and 1,200–900 cm^-1^. The dendrogram was constructed with Ward´s algorithm. The cut-off value of D = 6 is indicated with the dotted line.

By FT-IR spectroscopy we could also get insight into the effect of plasma exposure on the biofilm matrix. In particular, a chemical and structural disruption in carbohydrates was observed (Fig 9). The plasma-mediated damage to Psl could be monitored by an increase in the absorbance of the rhamnose characteristic functional groups (C-H in CH_3_ at 2,550 cm^-1^ in W2 and C-O-C at 1,043–44 cm^-1^ in W4) and of D-mannose and D-glucose, (C-O-C in W4). A fraction of these moieties, which are hidden in the Psl normal helical structure (Fig 9A) might be probably exposed and therefore enhance their FT-IR absorbance after plasma treatment. A multivariate analysis of the second derivative spectra in W4 (at C-H of CH_3_ band) effectively reflected the overall structural variations in the polysaccharide structure induced by 3 and 30 min of plasma treatment (Fig 9B). Three groups of spectra, one for each plasma exposure time, were clustered in the dendrogram. The most noticeable effects of plasma on the structure and chemical composition of matrix polysaccharides were observed after 3 min of treatment. The control group was at a spectral distance of D = 18 with respect to groups corresponding to 3 and 30 min of plasma treatment as shown in Fig 9C. Therefore, and in agreement with the microscopy observations, the effect of plasma on the matrix polysaccharides is evident already at short exposure times.

Our results differ from recent results reported by Khan et al. [78] who showed a gradual reduction in carbohydrates, proteins, lipids, and DNA contents when increasing plasma exposure time. However, it is important to remark that Kahn and co-workers rinse the samples after plasma treatment and thus, all the detached material is removed.

**Fig 10:**
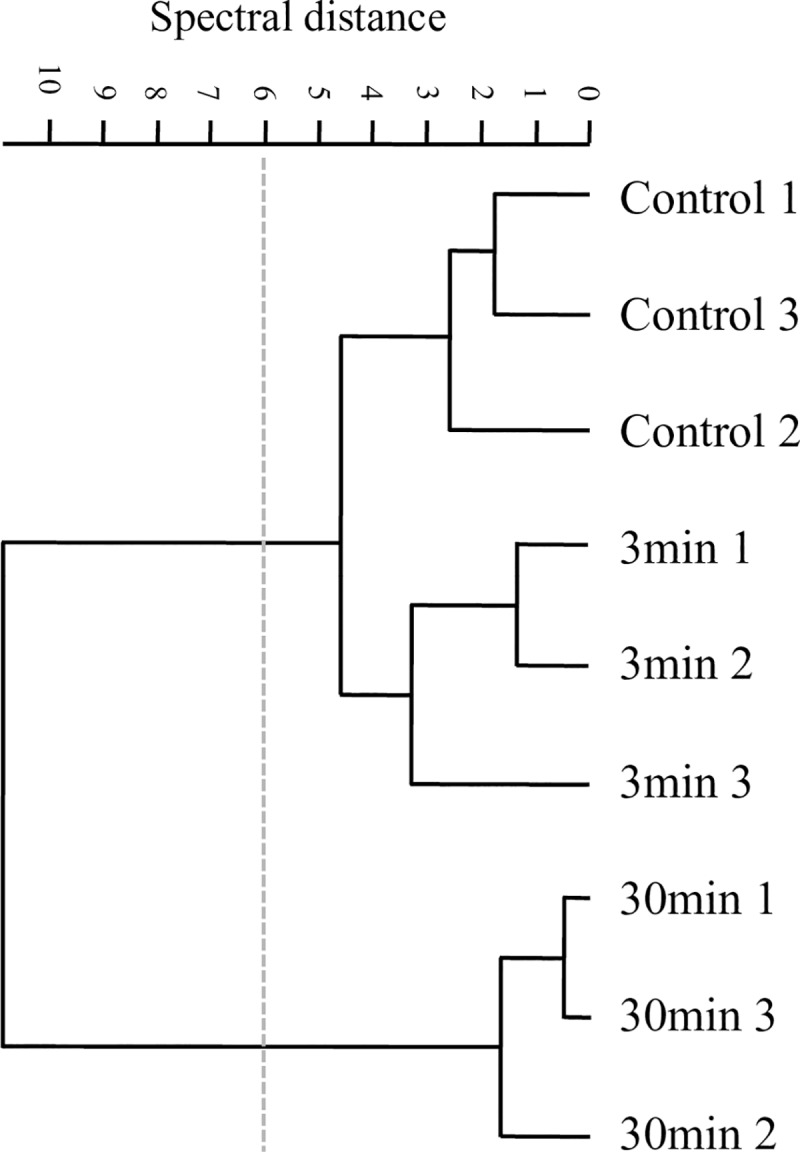
Plasma effect on DNA. Cluster analysis obtained with the 2^nd^ derivatives as input data using scaling to first range for spectral distance calculation in the spectral ranges 1,500–1,200 cm^-1^. Dendrogram was constructed with Ward´s algorithm. The cut-off value of D = 6 is indicated with the dotted line.

Regarding eDNA present in the matrix, it was previously reported that the photodamage of UV-light to DNA is not directly reflected in the intensity of the bands typically assigned to DNA such as 2,950, 1,963, 1,220, and 1,067 cm^-1^ [45]. Previous investigations showed that a high proportion of intracellular DNA is released from the cytoplasm due to cell membrane damage, preventing DNA-band analysis [78]. In addition, modifications in phosphate bands are also difficult to observe because of the numerous overlaps [79]. Nonetheless, it was possible to observe a noticeable general damage to DNA after 30 min of plasma exposure through changes in the features of the second derivative spectra in the 1,500–1,300 cm^-1^ region (W3) (Fig 10). These changes were particularly significant at 1,464, 1,396, 1,302 cm^-1^ peaks, which were previously assigned to photodamage to thymidine oligonucleotides [80].

In summary, through FT-IR we demonstrated that plasma treatment induces chemical and structural changes in both the biofilm matrix and the biomass. Those changes are more profound with longer plasma exposure times and affect biomass, lipids, carbohydrates, proteins, and eDNA.

### A model for plasma-mediated biofilm eradication

Plasma-mediated biofilm eradication has been investigated for more than a decade. Many of those studies were aimed at finding appropriate plasma sources and the best operation conditions to eradicate biofilms. Several mechanisms were hypothesized and a considerable amount of evidence showed morphological and physiological changes in the biofilm cells upon plasma treatment.

Biofilms cells are embedded in a protective matrix composed of extracellular polymeric substances. If plasma presumably damaged the cells, there should be an effect on the biofilm matrix as well. However, this effect has received less attention compared to the damage to cells. Although many contributions have highlighted the putative mechanism/s that might explain the interaction of the plasma reactive agents with the biofilm matrix, there is little evidence regarding the changes the matrix undergoes.

In this work, we have carried out a comprehensive study of the biofilm matrix and the structural and chemical changes the matrix and sessile cells go through upon plasma treatment. To our knowledge this is the first report providing detailed evidence of the variety of chemical and structural changes that occur mostly on the biofilm matrix but also on sessile cells as a consequence of the plasma treatment. In summary, we demonstrated that the amount of biofilm matrix is reduced and its structure is altered upon plasma treatment. We showed that eDNA plays a role in biofilm protection against plasma since the enzymatic degradation of the DNA results in a better and faster biofilm eradication. We also demonstrated for the first time, chemical and structural disruption in biofilm matrix carbohydrates with an increase in rhamnose, D-mannose, and D- glucose typically hidden in the Psl normal helical structure and seemingly exposed after plasma treatment. We showed DNA photodamage and a clear damage to sessile biomass due to significant changes in the protein secondary structure.

In addition, in a previous contribution we characterized our plasma source by optical emission spectroscopy and detected the presence of excited N_2_ bands and N_2_^+^ ions and the formation of O_3_, H_2_O_2_ and NO_3_^-^ in the plasma afterglow [41]. We then hypothesized that these reactive species might trigger lipid peroxidation and a chain reaction leading to the generation of lipid radicals. The oxidation of the matrix components might lead to the degradation and loss of the matrix structure allowing RONS to reach the cells. In this paper, we determined the presence of lipid oxidation products through FT-IR, thus confirming cell membrane lipid peroxidation as plasma exposure time increases.

Taking these results together, we propose a mechanism by which the reactive species and UV radiation in the plasma alters eDNA, carbohydrates, and other components of the biofilm matrix resulting in a less structured protective case and leading to more vulnerable cells. Our results are consistent with a matrix reduction due to the combined effect of UV radiation, RONS, and plasma ions. All these reactive agents might damage the eDNA-Psl fibers at short plasma exposure times and oxidize the rest of the matrix macromolecules. Considering the substantial contribution of the UV radiation to the biofilm eradication process and the lower penetration efficiency of this type of radiation, we propose that UV affects not only the upper layers of the biofilm matrix but also its protective eDNA, destabilizing or degrading the eDNA-Psl matrix network and opening an avenue for RONS and ions further action. RONS also contribute to DNA damage in synergism with UV. Afterwards, plasma may act directly on the sessile cells producing lysis due to the combination of oxidative stress, electrostatic changes, and electroporation. Sessile cells undergo sequential morphological and physiological changes, losing their cultivability although not their virulence to finally succumb to the treatment [18].

These results show the potential of the air-operated DBD plasma source and provide detailed evidence on the putative mechanism of plasma-mediated biofilm eradication.

## Supporting information

S1 Table Set of data points and statistical analysis for the biofilm matrix area, the sessile cells area, and the ration between both obtained from binary images of plasma-treated biofilms. The ratio corresponds to the black pixel area in the binary image of the biofilm matrix stained with Calcofluor White and the black pixel area in the binary image of the biofilm cells stained with SYTO9. Biofilms were treated with plasma for 0, 3, and 30 min prior to staining. Images were binarized as indicated in Fig 3.
(XLSX)

S2 Table Set of data points and statistical analysis for the semi-quantitative analysis of primary lipid oxidation products and final oxidation products. Primary and secondary lipid oxidation products were measured by spectral band areas at 1,740–45 cm^-1^ and 1,715–20 cm^-1^, respectively. Spectral band area values were obtained from the average spectra calculated at each plasma exposure time (0, 3, and 30 min).
(XLSX)

S1 Fig Reproducibility levels among biological replicates of *P*. *aeruginosa* biofilms for different plasma exposure time. Spectral distances (D) were calculated with normal to reprolevel method in the spectral windows 3,000–2,800 cm^−1^; 1,800–1,550 cm^−1^; 1,500–1,250 cm^−1^ and 1,200–900 cm^−1^, and dendrograms were obtained using Average Linkage (OPUS software 7.0, Bruker, Optics, Germany). (A) no plasma treatment, (B) 3 min plasma exposure time, (C) 30 min plasma exposure time.
(TIF)
