# Supplementary figures and images for: The Pseudomonas aeruginosa biofilm matrix and cells are drastically impacted by gas discharge plasma treatment: A comprehensive model explaining plasma-mediated biofilm eradication
Source: PLoS One. 2019 Jun 24;14(6):e0216817. doi: 10.1371/journal.pone.0216817 (PMC6590783; doi:10.1371/journal.pone.0216817)

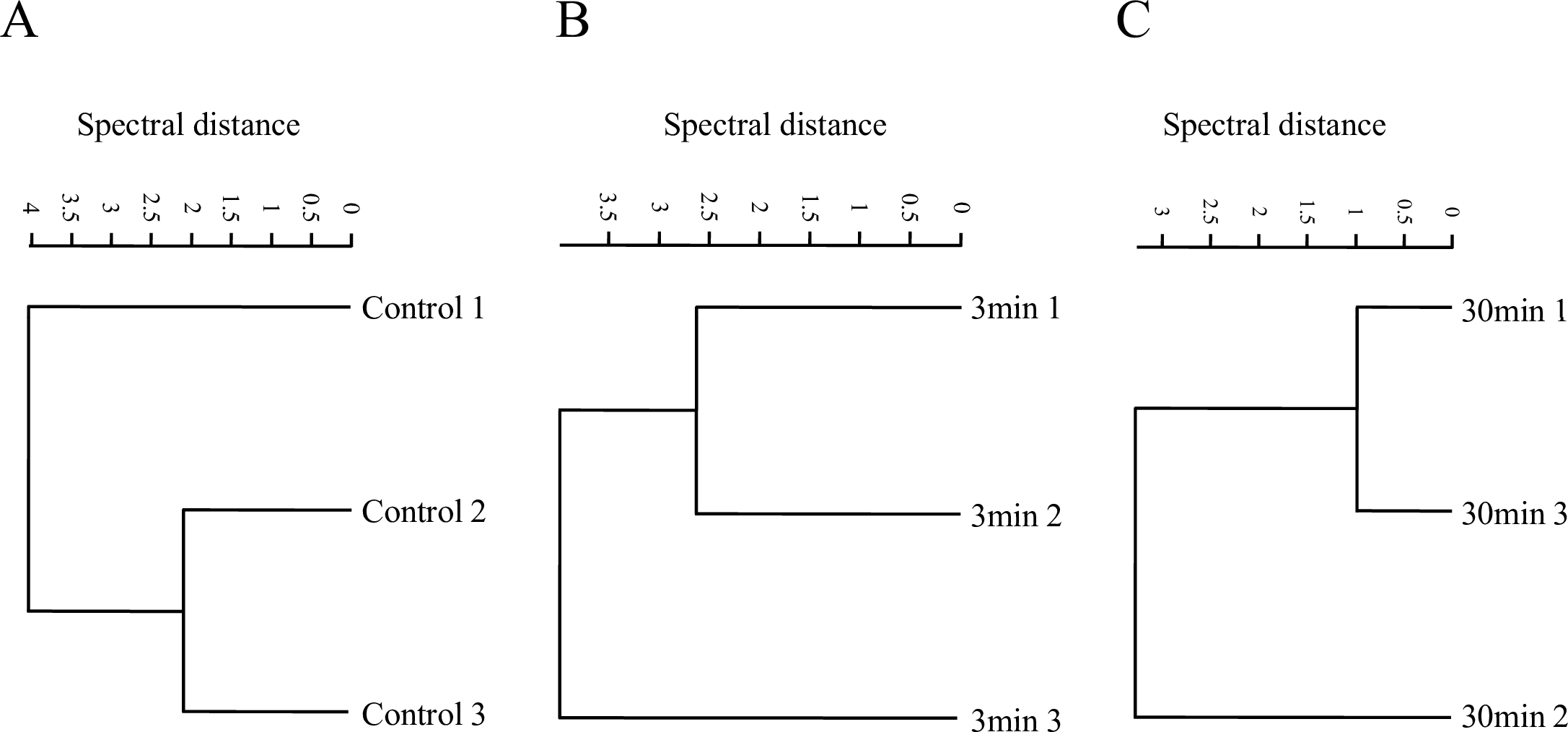

Supplement: S1 Fig — Spectral distances (D) were calculated with normal to reprolevel method in the spectral windows 3,000–2,800 cm−1; 1,800–1,550 cm−1; 1,500–1,250 cm−1 and 1,200–900 cm−1, and dendrograms were obtained using Average Linkage (OPUS software 7.0, Bruker, Optics, Germany). (A) no plasma treatment, (B) 3 min plasma exposure time, (C) 30 min plasma exposure time. (TIF) [file pone.0216817.s003.tif]
